# Treatment of dorsally dislocated distal radius fractures with individualized 3D printed bracing: an exploratory study

**DOI:** 10.1186/s41205-020-00075-4

**Published:** 2020-08-18

**Authors:** H. M. J. Janzing, S. A. M. Bessems, M. A. P. Ligthart, E. M. M. Van Lieshout, H. P. Theeuwes, D. G. Barten, M. H. J. Verhofstad

**Affiliations:** 1 Department of Surgery, VieCuri Medical Center, Venlo, The Netherlands; 2 Trauma Research Unit Department of Surgery, Erasmus MC, University Medical Center Rotterdam, Rotterdam, The Netherlands; 3 Department of Surgery, Elisabeth-Tweesteden Hospital, Tilburg, The Netherlands; 4 Department of Emergency Medicine, VieCuri Medical Center, Venlo, The Netherlands

## Abstract

**Background:**

The aim of this work was to develop a three-dimensionally (3D) printed brace for the acute treatment of dorsally dislocated and correctly reduced distal radius fractures (DRF). The hypothesis was that a brace shaped to the mirror image of the contralateral (non-fractured) wrist will have an optimal anatomical fit, resulting in improved comfort and lower rates of secondary fracture displacement.

**Method:**

Validation: the circumference of both wrists and comfort of the brace were studied in healthy volunteers and effectiveness of the brace was evaluated in an ex vivo fracture model.

Clinical study: the brace was tested for comfort and effectiveness in patients with a well reduced unstable DRF.

**Results:**

Validation: the circumference of both wrists may be different, the brace retained the reduction in the ex vivo fracture model and was well tolerated in the volunteers.

Clinical study: in DRF patients comfort scores were lower and pain scores higher compared to the healthy volunteers. After 3 and 5 weeks all patients were independent in ADL according to the Katz-index. Posttraumatic swelling subsided in the first week. In two of the five patients secondary fracture dislocation occurred.

**Conclusions:**

Treatment of a dislocated DRF in the acute setting (day one) with a custom-made 3D printed brace, anatomically modelled from a 3D scan of the contralateral wrist, is possible. Difference between both wrists and posttraumatic swelling must be adapted for. The high rate of secondary fracture displacement led to early discontinuation of the study and a small sample size.

**Trial registration:**

Name of the registry: ClinicalTrials.Gov

Trial registration number: NCT03848702

Date of registration: 02/21/2019, retrospectively registered

## Background

Distal radius fractures (DRF) are increasingly common [1] and have a high functional impact. Closed reposition and non-operative treatment with casting is complicated by secondary displacement in up to 75% of cases [2]. Secondary displacement refers to displacement of a fracture after manipulation to an anatomic position.

Three-dimensional (3D) printing is an emerging technique that allows for individualized modeling and production. Some start-ups are exploring the development and implementation of 3D printed braces for fracture treatment. Chen et al. published their clinical experience in a group of 10 patients [3]. Their use of the fractured limb as a template presumes a non-dislocated or perfectly reduced fracture. They started with a classical plaster cast and changed to a 3D printed brace after 1 week when swelling had subsided. No loss of reduction or serious complications were observed. We did not find previous studies using a 3D printed brace for the acute or initial treatment of dislocated DRF.

The aim of this project was to develop a custom-made 3D printed brace that could be used in the acute treatment of dorsally dislocated and correctly reduced DRFs. Focus was on dislocated fractures because non-dislocated fractures are easily treated using less complicated techniques. The hypothesis was that by using the contour of the non-fractured contralateral wrist as a (mirrored) template, a brace with an optimal anatomical fit could be made. With this individualized brace we aimed to improve comfort and reduce secondary fracture displacement.

## Materials and methods

### Validation

Before the clinical study three validation studies were performed:

#### Study of the comparability of both wrists in healthy volunteers

In order to use the contour of the contralateral wrist as a template both wrists must be comparable. We planned to compare the wrists in at least 100 volunteers who were 18 years or older and had no former treatment for a wrist fracture. After informed consent was obtained circumference of wrists was measured in millimetres (mm), distally from the styloid at the base of the hand with the wrist in neutral position. Since either side could have the largest circumference, the difference in circumferences of the largest versus that of the smallest wrist was tested with a paired t-test (with *p* ≤ 0,05 considered as statistically significant).

#### Evaluation of the brace in an ex vivo model

The brace was tested in an ex vivo model using six AnubiFiX®™ (Erasmus MC, Rotterdam, The Netherlands) embalmed human specimen. These anatomical specimen remain flexible [4]. The DRF was simulated with the model published by Baumbach et al. [5] modified by retaining the soft tissues: a wedge osteotomy of 10 mm dorsal/1 mm volar was made 8 mm/12 mm proximal to the dorsal/volar apex of the articular surface.

**Fig. 1:**
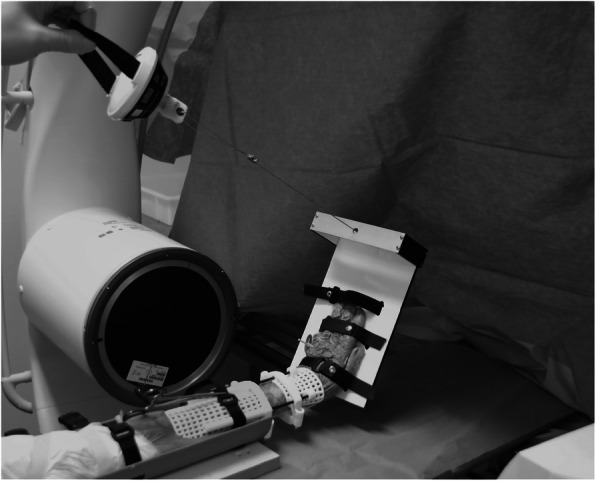
The ex vivo testing of the brace

The dislocating forces were simulated using the model described by Theeuwes et al. [4]: with the forearm fixated, a force of 20 Nm was applied to the hand, first in dorsal and then in radial direction. Displacement of the fracture (osteotomy) was radiologically assessed without brace. Then the brace was applied, the same dislocating force was used and the fracture position was radiologically redetermined (Fig. 1). Fracture position was evaluated by radiological evaluation using the criteria of the ‘Dutch Guideline for Distal Radius Fractures’ [6]. A fracture was considered displaced if any of the following conditions applied:

- Dorsal tilt > 15 degrees on lateral X-ray
- Volar tilt > 20 degrees on lateral X-ray
- Shortening > 5 mm pertaining to the ulna in PA direction
- Intraarticular Step-off ≥2 mm
- Radial inclination < 15 degrees in AP X-ray
- Subluxation of the lunate

#### Study of comfort in healthy volunteers

To test the brace for comfort the brace was applied to 10 healthy volunteers for 7 days. All volunteers were aged 50 years or older, had no DRF and were included between the 20th of July until the 26th of July 2017. Exclusion criteria were restrictions in activities of daily living (ADL); pre-existing anatomical deformation of ipsi- or contralateral wrist; impaired wrist function or a known allergy for polylactic acid (PLA).

Computer randomization was performed to obtain bracing of 5 dominant and 5 non-dominant wrists.

The contralateral wrist was scanned with a Structure optical 3D scanner (Occipital, Inc. San Francisco, US) with the volunteer lying supine with flexed elbow, forearm in vertical position, neutral wrist extension and neutral pro-supination and with traction on the second and third fingers applied through finger traps. The scan was then digitally mirrored, the position of the wrist scan was aligned with the position of the reference brace and the pads and reinforcements of the reference brace were adapted to the 3D scan using Blender open source software.

**Fig. 2:**
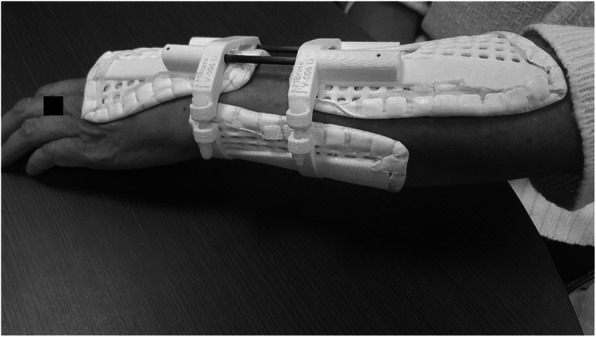
Brace with three point fixation

The brace design was adapted for any (left/right) difference in volunteer’s wrist circumference. Compared to the original design (Fig. 1) we lengthened the distal dorsal brace pad to avoid pain at the back of the hand during wrist extension (Fig. 2).

The three brace-pads were printed with a fused deposition modeling (FDM) printer (Wanhao duplicator I3, Wanhao, Zheiang, China) with PLA filament (Polymaker Polymax PLA, 1,7 mm, Polymaker, Utrecht, Netherlands). Aquacast® lining (Aquacast Liner LLC, Newar DE19702) was added. The two dorsal pads were connected by carbon rods, the dorsal and volar parts by polyethylene screws.

The brace was fitted on day zero.

The primary outcome measure was a 100 mm Visual Analog Scale (VAS) for wearing comfort, with 0 mm being extremely uncomfortable and 100 mm being extremely comfortable. A VAS is a measurement instrument for subjective characteristics or attitudes that cannot be directly measured, like pain or comfort. When responding to a VAS item, respondents specify their level of agreement to a statement by indicating a position along a continuous line between two end-points [7].

The secondary outcome measures were:

- pain caused by the brace scored on a 100 mm VAS where 0 mm implies no pain and 100 mm implies the worst possible pain;
- dependency during ADL measured by the Katz-index (inquires about limitations in washing, clothing, indoor transfers, toilet visit, continence and eating, where A is independent for all items and G is dependent for all items) [8];
- adverse reactions like skin pressure, skin irritation/redness, sensory abnormalities, or device-related problems.

Both primary and secondary outcome measures were collected on day one, three and seven. Skin inspection and assessment for adverse reactions was done in an outpatient clinic at day seven or in case of earlier complaints of discomfort on day one or three.

### Clinical study

Finally, the brace was tested for comfort and effectiveness in patients with a well reduced unstable DRF. For this pilot study we planned to include 10 patients, aged 50 years or older, diagnosed at the Emergency Department (ED) with an acute DRF (OTA fracture type 23 A, B, or C with dorsal dislocation) [9] with acceptable fracture reduction in the period between the 8th of January and the 28th of March 2018. Exclusion criteria were pre-existing restriction in ADL, pre-existing anatomical deviation of the ipsi- or contralateral wrist; pre-existing impaired wrist function; known allergy for polylactic acid; pathological, recurrent or open fractures; bone disorders (excluding osteoporosis) and additional traumatic injuries affecting treatment and prognosis of the DRF.

On the day of presentation at the ED, closed reduction was performed. After closed reduction, the wrist circumference of both arms was measured and used to customize the brace. The limb was temporarily splinted with a plaster cast and acceptability of reduction was assessed radiologically using the criteria of the ‘Dutch Guideline for Distal Radius Fractures’ [6]. If the reduction was acceptable and informed consent was provided the patient was included in the study. The contralateral arm was scanned. The scanning, modeling and printing procedure was the same as described for the volunteer study. The brace was fitted on the first working day following enrolment. Treatment duration was 5 weeks.

Outcome measures were collected on day two or three and at one, two, and 5 weeks after fitting. Radiological and clinical assessment was done in an outpatient clinic at one, 2 and 5 weeks. Outcome measures included secondary displacement and all outcome measures previously listed for the study in healthy volunteers. Secondary displacement was determined radiologically as described in the ‘Dutch Guideline for Distal Radius Fractures’ as mentioned earlier.

The study in healthy volunteers and the clinical study were approved by the Medical Research Ethics Committee Erasmus MC, Rotterdam, The Netherlands (Ref. No. NL88 61,002.078.17). All participants provided written informed consent prior to inclusion in the study.

## Results

### Validation

#### Study of the comparability of both wrists in healthy volunteers

Measurements of both wrists were compared in 118 healthy volunteers. Mean age was 62 years (range 22–100 years) and 62% of participants was female. The mean absolute difference in circumference between contralateral wrists was 2,9 ± 3,9 mm (range 0–20 mm). The paired t-test indicated that the circumference of the largest wrist was statistically significantly different from that of the contralateral wrist (*p* < 0.001). A difference in wrist circumference of 5 mm or more was found in 18% of the volunteers.

#### Evaluation of the brace in an ex vivo model

Radiographic assessment of the fracture model in the AnubiFiX®™ fixated arms showed a dislocation in radial inclination in all six osteotomies. Therefore, all fractures met the criteria for a dislocated fracture. Application of the brace resulted in a good position of the fracture, which was retained during application of force in all arms (Table 1).

**Table 1:** Ex vivo study: radiological measurements with brace

Arm	Radial inclination	Volar inclination	Ulnar variance
nr	AP X-ray, degrees	Lat. X -ray, degrees	AP X-ray, mm
**1**	23	0	0
**2**	23	15	3
**3**	20	12	-1
**4**	20	11	1
**5**	16	0	0
**6**	19	10	1

#### Study of comfort in healthy volunteers

Ten volunteers, with a mean age of 58 ± 6 years, participated in the volunteer study. Six of them were female. All volunteers had a right-hand dominance, resulting in the same number of braces fitted to the left and right forearm. The mean comfort score during the follow-up period was 80 ± 19 mm and the mean VAS scores for pain during all activities was 6 ± 11 mm. The Katz-index was A for all volunteers at all moments: this means that none of the volunteers were restricted in ADL in any of the categories defined in the Katz-index. In two volunteers minor skin problems were noted on day 7: a small blister of 1 cm in diameter on the volar wrist in one patient and a small superficial scrape on the dorsum of the ulnar head in the other. No sensory abnormalities of the median, ulnar, or radial nerve were noted.

### Clinical study

**Fig. 3:**
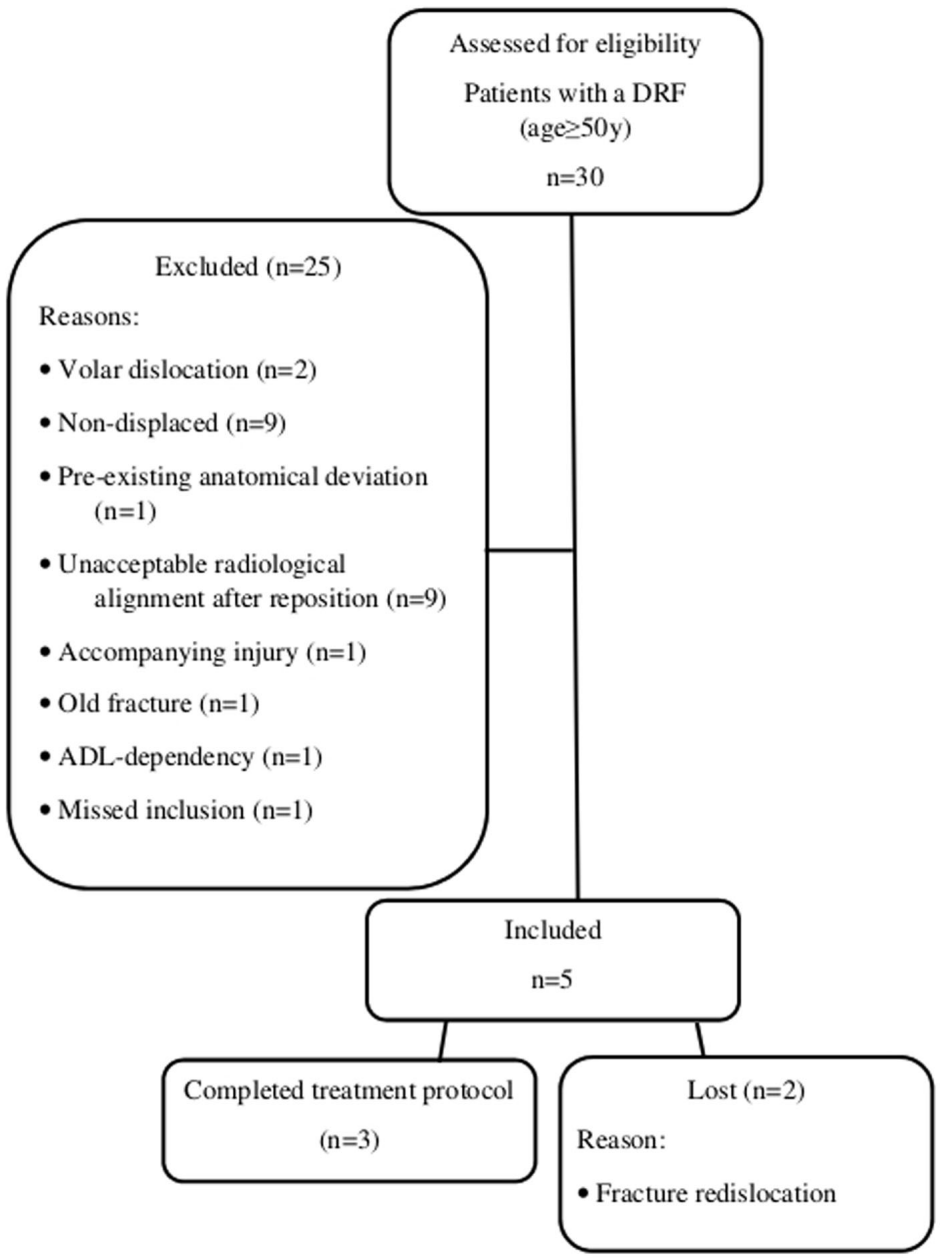
Flow diagram of the inclusions in the clinical study

Over the course of the study 30 patients with a DRF were assessed for eligibility at the ED of which 25 were excluded. Figure 3 shows the flow chart of this part of the study. After fracture reduction at the ED the mean difference in wrist circumference between the injured and uninjured arm was 13,2 ± 6,9 mm. During treatment the brace had to be tightened as swelling reduced to prevent a loose fit. In all patients swelling had largely subsided after the 1st week. Figure 2 shows the brace model used in the clinical patients. The dorsal and volar parts are connected by screws which can be tightened as swelling decreases over time. Three out of five patients completed the treatment protocol consisting of 5 weeks of brace immobilisation. The reason for switching treatment methods in the other two patients was secondary fracture displacement after 1 week. Both patients underwent open reduction and internal fixation.

The comfort scores are listed in Table 2. VAS scores for pain during rest and during daily activities are listed in Table 3. Katz scores are listed in Table 4. During the first weeks assistance was predominantly needed with bathing and preparing meals. After 3 and 5 weeks all patients were independent in ADL according to the Katz-index (score A). No sensory abnormalities of the median, ulnar or radial nerve were noted. Patient 3 suffered from a pressure point on the ulnar styloid (red discoloration and pain without skin necrosis).

**Table 2:** Comfort VAS in mm

Comfort VAS	2–3 days	1 wk	3 wks	5 wks
**Patient 1**	70	80	90	90
**Patient 2**	70			
**Patient 3**	60	50	10	10
**Patient 4**	100	80	90	90
**Patient 5**	70	80

**Table 3:** Pain VAS in mm at rest and during daily activities

Pain VAS rest/ADL	2–3 days	1 wk	3 wks	5 wks
Patient 1	0/60	0/60	0/70	0/0
Patient 2	60/missing			
Patient 3	65/80	45/80	80/missing	70/90
Patient 4	90/90	0/0	0/0	0/0
Patient 5	30/30	0/20

**Table 4:** Katz scores

Katz score	2–3 days	1 wk	3 wks	5 wks
**Patient 1**	D	B	A	A
**Patient 2**	A			
**Patient 3**	B	B	A	A
**Patient 4**	B	B	A	A
**Patient 5**	A	A		

After 5 weeks the three remaining patients showed acceptable radiological alignment according to the criteria of the ‘Dutch Guideline for Distal Radius Fractures’.

## Discussion

Differences between the circumferences of both wrists may be important and should be accounted for when using the mirrored scan of the contralateral wrist to produce a brace. The clinical study showed that direct posttraumatic swelling is even more important. This swelling had largely subsided after the 1st week. Tightening of the brace was needed in this period to avoid a loose fit.

Our ex vivo model resulted in a reproducible dislocated distal radius fracture. The brace corrected the fracture to an acceptable position in all specimen.

Comfort scores in healthy volunteers were good and pain scores were low. All volunteers had a maximal Katz score meaning there were no limitations in ADL. Two volunteers had minor skin problems without resulting pain. No severe side effects were noted. A literature search was performed and resulted in no comparative data.

In the clinical study comfort scores were lower and pain scores higher compared to the healthy volunteers as might be expected with a fracture. Patient three reported high pain and poor comfort scores caused by a pressure point on the dorsum of the ulnar head, the same anatomical spot as in one of the volunteers. A possible cause for this pressure point is the increased prominence of the ulnar head during pronation of the wrist (as in typewriting). The same pressure problem on the ulnar head was described by Chen et al. [3]. Patients were initially restricted in ADL but independent after 3 and 5 weeks. Although functional decline and restrictions in ADL have been reported in older adults with distal radial fractures [10, 11] we found no literature about early ADL restrictions during cast or brace treatment to compare with. The customized brace resulted in acceptable comfort and we encountered no serious complications other than secondary fracture displacement in two patients.

In an earlier preclinical study we replaced the custom made 3D printed brace by a similar “confection brace”. We 3D printed similar braces with three-point fixation in eight different stock sizes, based on the 3D scans and measurements of 50 healthy volunteers. This “confection-brace” failed to produce a comfortable fit in most volunteers (unpublished data).

Two secondary fracture displacements in five patients is comparable with the results of non-operative treatment of DRF in the literature [12]. Prognostic factors for redisplacement after initial closed reduction are greater initial displacement and age [13]. So it is not unexpected to see a high rate of redisplacement in our target group: the older patient with severely dislocated DRF. A Cochrane review on non-operative interventions found no conclusive evidence for the superiority of any immobilisation method in distal radius fractures [14]. The Aberdeen Colle’s fracture brace is based on three-point fixation and good clinical results has been published [15]. Although insufficient stability was not mentioned as a motivation for abandoning further development, a patent application combining functional bracing with K-wires suggests that stability might have been a problem [16]. Chen et al. did report no loss of reposition in 10 patients with a DRF treated with a week traditional plaster cast followed by treatment in a 3d printed brace [3]. We hoped for the same extraordinary result but could not reproduce it. Possibly Chen et al. excluded patients with unstable DRFs.

As we explained in the background section we hoped that a personalized brace would result in less secondary fracture displacement than classic non-operative treatment. This was not confirmed and made us prematurely stop patient inclusion.

The subjective experience in the ex vivo study, volunteer study and clinical study was that the brace showed a good fit with adequate three-point fixation without major pressure problems. In the ex vivo model we confirmed the adequacy of fracture reduction. Nevertheless two secondary fracture displacements were seen in five patients in the clinical study. Our hypothesis about the failure of the brace in preventing secondary fracture displacement is that despite providing a good anatomical fit, it cannot compensate for the dislocation forces caused by the brachioradial and carpal extensor muscles. This would explain the difference between the results of the ex vivo and clinical study and the high rate of secondary displacement of unstable DRF in any non-operative treatment.

The major weakness of this study is the small sample size and the remaining question whether better results could be achieved with an improved brace. Our experience and hypothesis about the failure of the brace is why we decided not to persevere in this work.

## Conclusions

Possible wrist circumference difference and posttraumatic swelling must be adapted for and most swelling subsides during the first week.

The custom-made 3D printed brace effectively preserves reduction of a dislocated DRF in the ex vivo model.

Healthy volunteers experienced good comfort, minimal pain and no restrictions in ADL while wearing the brace.

Treatment of a dislocated DRF in the acute setting (day one) with a custom-made 3D printed brace, anatomically modelled from a 3D scan of the contralateral wrist is possible.

The custom-made brace failed to prevent secondary fracture displacement in two of the five patients. This lead to early discontinuation of the study and a small sample size.

## Data Availability

The datasets used and/or analysed during the current study are available from the corresponding author on reasonable request.
